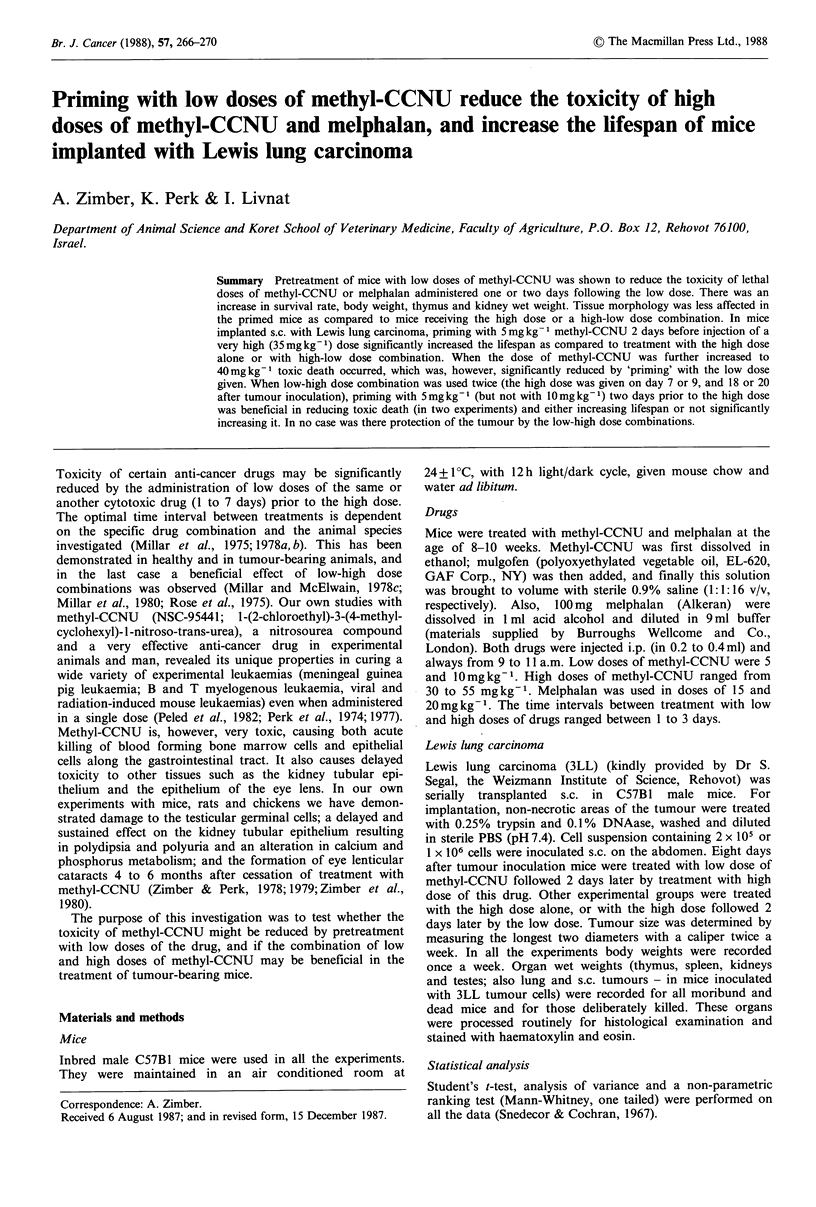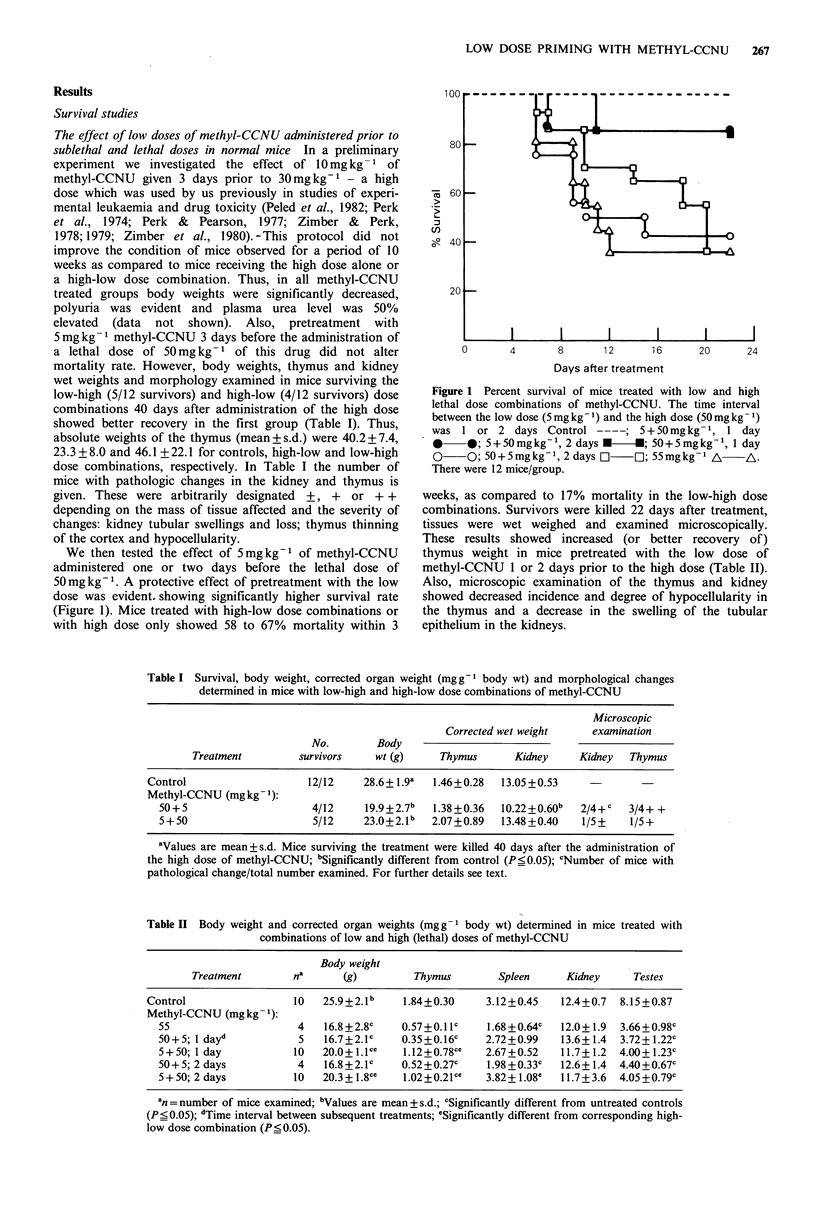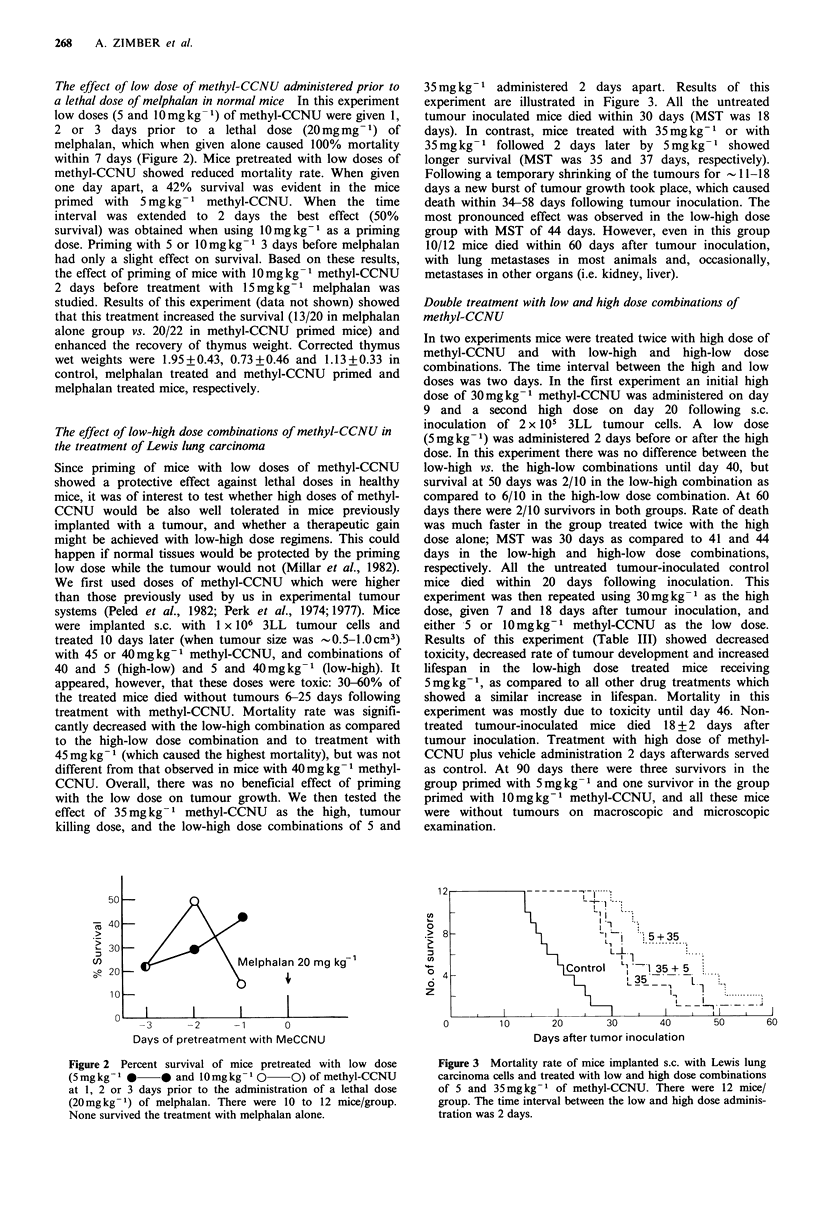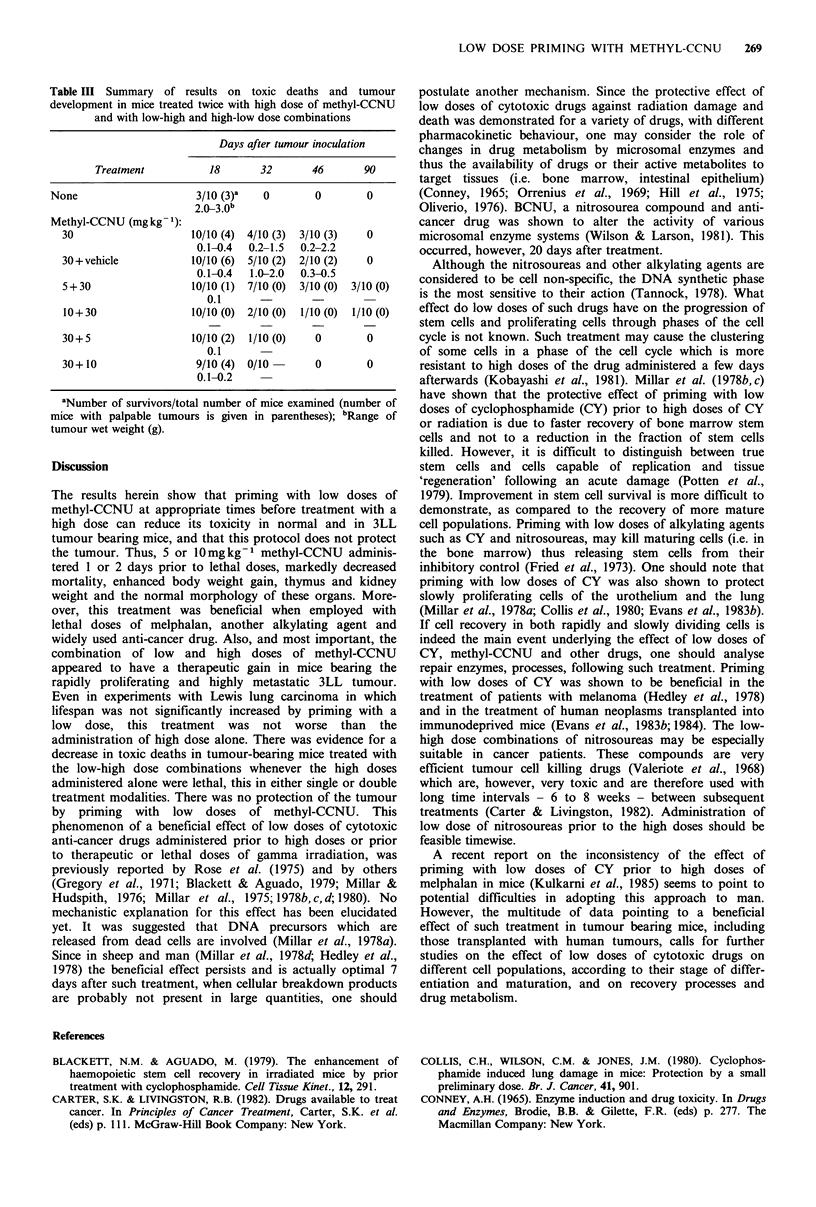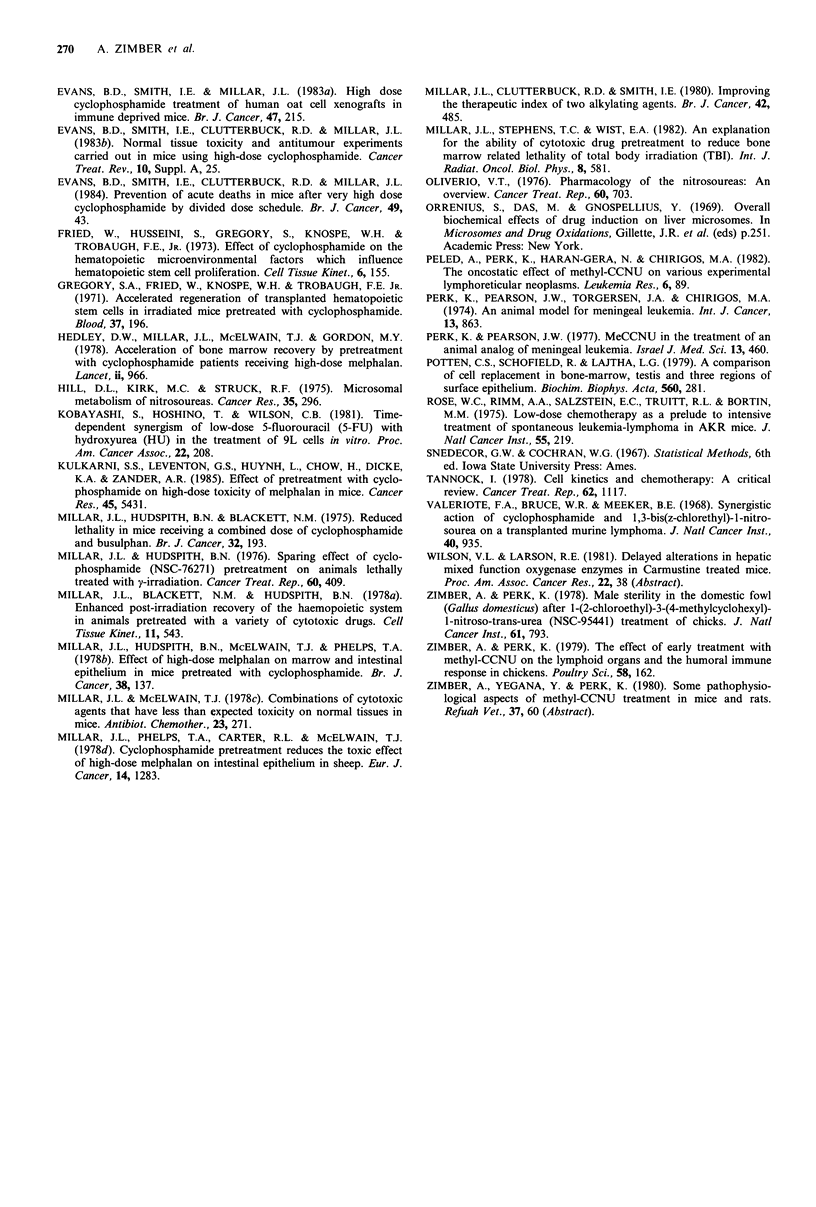# Priming with low doses of methyl-CCNU reduce the toxicity of high doses of methyl-CCNU and melphalan, and increase the lifespan of mice implanted with Lewis lung carcinoma.

**DOI:** 10.1038/bjc.1988.57

**Published:** 1988-03

**Authors:** A. Zimber, K. Perk, I. Livnat

**Affiliations:** Department of Animal Science, Faculty of Agriculture, Israel.

## Abstract

Pretreatment of mice with low doses of methyl-CCNU was shown to reduce the toxicity of lethal doses of methyl-CCNU or melphalan administered one or two days following the low dose. There was an increase in survival rate, body weight, thymus and kidney wet weight. Tissue morphology was less affected in the primed mice as compared to mice receiving the high dose or a high-low dose combination. In mice implanted s.c. with Lewis lung carcinoma, priming with 5 mg kg-1 methyl-CCNU 2 days before injection of a very high (35 mg kg-1) dose significantly increased the lifespan as compared to treatment with the high dose alone or with high-low dose combination. When the dose of methyl-CCNU was further increased to 40 mg kg-1 toxic death occurred, which was, however, significantly reduced by 'priming' with the low dose given. When low-high dose combination was used twice (the high dose was given on day 7 or 9, and 18 or 20 after tumour inoculation), priming with 5 mg kg-1 (but not with 10 mg kg-1) two days prior to the high dose was beneficial in reducing toxic death (in two experiments) and either increasing lifespan or not significantly increasing it. In no case was there protection of the tumour by the low-high dose combinations.


*Br. J. Cancer* (1988), **57**, 266–270 © The Macmillan Press Ltd., 1988

### Priming with low doses of methyl-CCNU reduce the toxicity of high doses of methyl-CCNU and melphalan, and increase the lifespan of mice implanted with Lewis lung carcinoma

A. Zimber, K. Perk & I. Livnat

*Department of Animal Science and Koret School of Veterinary Medicine, Faculty of Agriculture, P.O. Box 12, Rehovot 76100, Israel.*

**Summary** Pretreatment of mice with low doses of methyl-CCNU was shown to reduce the toxicity of lethal doses of methyl-CCNU or melphalan administered one or two days following the low dose. There was an increase in survival rate, body weight, thymus and kidney wet weight. Tissue morphology was less affected in the primed mice as compared to mice receiving the high dose or a high-low dose combination. In mice implanted s.c. with Lewis lung carcinoma, priming with $5\,mg\,kg^{-1}$ methyl-CCNU 2 days before injection of a very high ($35\,mg\,kg^{-1}$) dose significantly increased the lifespan as compared to treatment with the high dose alone or with high-low dose combination. When the dose of methyl-CCNU was further increased to $40\,mg\,kg^{-1}$ toxic death occurred, which was, however, significantly reduced by 'priming' with the low dose given. When low-high dose combination was used twice (the high dose was given on day 7 or 9, and 18 or 20 after tumour inoculation), priming with $5\,mg\,kg^{-1}$ (but not with $10\,mg\,kg^{-1}$) two days prior to the high dose was beneficial in reducing toxic death (in two experiments) and either increasing lifespan or not significantly increasing it. In no case was there protection of the tumour by the low-high dose combinations.

Toxicity of certain anti-cancer drugs may be significantly reduced by the administration of low doses of the same or another cytotoxic drug (1 to 7 days) prior to the high dose. The optimal time interval between treatments is dependent on the specific drug combination and the animal species investigated (Millar *et al.*, 1975; 1978*a*, *b*). This has been demonstrated in healthy and in tumour-bearing animals, and in the last case a beneficial effect of low-high dose combinations was observed (Millar and McElwain, 1978*c*; Millar *et al.*, 1980; Rose *et al.*, 1975). Our own studies with methyl-CCNU (NSC-95441; 1-(2-chloroethyl)-3-(4-methylcyclohexyl)-1-nitroso-trans-urea), a nitrosourea compound and a very effective anti-cancer drug in experimental animals and man, revealed its unique properties in curing a wide variety of experimental leukaemias (meningeal guinea pig leukaemia; B and T myelogenous leukaemia, viral and radiation-induced mouse leukaemias) even when administered in a single dose (Peled *et al.*, 1982; Perk *et al.*, 1974; 1977). Methyl-CCNU is, however, very toxic, causing both acute killing of blood forming bone marrow cells and epithelial cells along the gastrointestinal tract. It also causes delayed toxicity to other tissues such as the kidney tubular epithelium and the epithelium of the eye lens. In our own experiments with mice, rats and chickens we have demonstrated damage to the testicular germinal cells; a delayed and sustained effect on the kidney tubular epithelium resulting in polydipsia and polyuria and an alteration in calcium and phosphorus metabolism; and the formation of eye lenticular cataracts 4 to 6 months after cessation of treatment with methyl-CCNU (Zimber & Perk, 1978; 1979; Zimber *et al.*, 1980).

The purpose of this investigation was to test whether the toxicity of methyl-CCNU might be reduced by pretreatment with low doses of the drug, and if the combination of low and high doses of methyl-CCNU may be beneficial in the treatment of tumour-bearing mice.

### Materials and methods

### *Mice*

Inbred male C57B1 mice were used in all the experiments. They were maintained in an air conditioned room at $24\pm1°C$, with 12h light/dark cycle, given mouse chow and water *ad libitum*.

### *Drugs*

Mice were treated with methyl-CCNU and melphalan at the age of 8–10 weeks. Methyl-CCNU was first dissolved in ethanol; mulgofen (polyoxyethylated vegetable oil, EL-620, GAF Corp., NY) was then added, and finally this solution was brought to volume with sterile 0.9% saline (1:1:16 v/v, respectively). Also, 100 mg melphalan (Alkeran) were dissolved in 1 ml acid alcohol and diluted in 9 ml buffer (materials supplied by Burroughs Wellcome and Co., London). Both drugs were injected i.p. (in 0.2 to 0.4 ml) and always from 9 to 11 a.m. Low doses of methyl-CCNU were 5 and $10\,mg\,kg^{-1}$. High doses of methyl-CCNU ranged from 30 to 55 $mg\,kg^{-1}$. Melphalan was used in doses of 15 and $20\,mg\,kg^{-1}$. The time intervals between treatment with low and high doses of drugs ranged between 1 to 3 days.

### *Lewis lung carcinoma*

Lewis lung carcinoma (3LL) (kindly provided by Dr S. Segal, the Weizmann Institute of Science, Rehovot) was serially transplanted s.c. in C57B1 male mice. For implantation, non-necrotic areas of the tumour were treated with 0.25% trypsin and 0.1% DNAase, washed and diluted in sterile PBS (pH 7.4). Cell suspension containing $2\times10^5$ or $1\times10^6$ cells were inoculated s.c. on the abdomen. Eight days after tumour inoculation mice were treated with low dose of methyl-CCNU followed 2 days later by treatment with high dose of this drug. Other experimental groups were treated with the high dose alone, or with the high dose followed 2 days later by the low dose. Tumour size was determined by measuring the longest two diameters with a caliper twice a week. In all the experiments body weights were recorded once a week. Organ wet weights (thymus, spleen, kidneys and testes; also lung and s.c. tumours – in mice inoculated with 3LL tumour cells) were recorded for all moribund and dead mice and for those deliberately killed. These organs were processed routinely for histological examination and stained with haematoxylin and eosin.

### *Statistical analysis*

Student's *t*-test, analysis of variance and a non-parametric ranking test (Mann-Whitney, one tailed) were performed on all the data (Snedecor & Cochran, 1967).

Correspondence: A. Zimber.
Received 6 August 1987; and in revised form, 15 December 1987.

### Results

### *Survival studies*

*The effect of low doses of methyl-CCNU administered prior to sublethal and lethal doses in normal mice* In a preliminary experiment we investigated the effect of $10\,mg\,kg^{-1}$ of methyl-CCNU given 3 days prior to $30\,mg\,kg^{-1}$ – a high dose which was used by us previously in studies of experimental leukaemia and drug toxicity (Peled *et al.*, 1982; Perk *et al.*, 1974; Perk & Pearson, 1977; Zimber & Perk, 1978; 1979; Zimber *et al.*, 1980). This protocol did not improve the condition of mice observed for a period of 10 weeks as compared to mice receiving the high dose alone or a high-low dose combination. Thus, in all methyl-CCNU treated groups body weights were significantly decreased, polyuria was evident and plasma urea level was 50% elevated (data not shown). Also, pretreatment with $5\,mg\,kg^{-1}$ methyl-CCNU 3 days before the administration of a lethal dose of $50\,mg\,kg^{-1}$ of this drug did not alter mortality rate. However, body weights, thymus and kidney wet weights and morphology examined in mice surviving the low-high (5/12 survivors) and high-low (4/12 survivors) dose combinations 40 days after administration of the high dose showed better recovery in the first group (Table I). Thus, absolute weights of the thymus (mean ± s.d.) were 40.2 ± 7.4, 23.3 ± 8.0 and 46.1 ± 22.1 for controls, high-low and low-high dose combinations, respectively. In Table I the number of mice with pathologic changes in the kidney and thymus is given. These were arbitrarily designated ±, + or ++ depending on the mass of tissue affected and the severity of changes: kidney tubular swellings and loss; thymus thinning of the cortex and hypocellularity.

We then tested the effect of $5\,mg\,kg^{-1}$ of methyl-CCNU administered one or two days before the lethal dose of $50\,mg\,kg^{-1}$. A protective effect of pretreatment with the low dose was evident, showing significantly higher survival rate (Figure 1). Mice treated with high-low dose combinations or with high dose only showed 58 to 67% mortality within 3 weeks, as compared to 17% mortality in the low-high dose combinations. Survivors were killed 22 days after treatment, tissues were wet weighed and examined microscopically. These results showed increased (or better recovery of) thymus weight in mice pretreated with the low dose of methyl-CCNU 1 or 2 days prior to the high dose (Table II). Also, microscopic examination of the thymus and kidney showed decreased incidence and degree of hypocellularity in the thymus and a decrease in the swelling of the tubular epithelium in the kidneys.

**Figure 1** Percent survival of mice treated with low and high lethal dose combinations of methyl-CCNU. The time interval between the low dose ($5\,mg\,kg^{-1}$) and the high dose ($50\,mg\,kg^{-1}$) was 1 or 2 days Control ----; $5+50\,mg\,kg^{-1}$, 1 day ●——●; $5+50\,mg\,kg^{-1}$, 2 days ■——■; $50+5\,mg\,kg^{-1}$, 1 day O——O; $50+5\,mg\,kg^{-1}$, 2 days □——□; $55\,mg\,kg^{-1}$ △——△. There were 12 mice/group.

**Table I** Survival, body weight, corrected organ weight ($mg\,g^{-1}$ body wt) and morphological changes determined in mice with low-high and high-low dose combinations of methyl-CCNU

| Treatment | No. survivors | Body wt (g) | Corrected wet weight | | Microscopic examination | |
|---|---|---|---|---|---|---|
| | | | Thymus | Kidney | Kidney | Thymus |
| Control | 12/12 | 28.6 ± 1.9[a] | 1.46 ± 0.28 | 13.05 ± 0.53 | — | — |
| Methyl-CCNU ($mg\,kg^{-1}$): | | | | | | |
| 50 + 5 | 4/12 | 19.9 ± 2.7[b] | 1.38 ± 0.36 | 10.22 ± 0.60[b] | 2/4+[c] | 3/4++ |
| 5 + 50 | 5/12 | 23.0 ± 2.1[b] | 2.07 ± 0.89 | 13.48 ± 0.40 | 1/5± | 1/5+ |

[a]Values are mean ± s.d. Mice surviving the treatment were killed 40 days after the administration of the high dose of methyl-CCNU; [b]Significantly different from control ($P \leq 0.05$); [c]Number of mice with pathological change/total number examined. For further details see text.

**Table II** Body weight and corrected organ weights ($mg\,g^{-1}$ body wt) determined in mice treated with combinations of low and high (lethal) doses of methyl-CCNU

| Treatment | n[a] | Body weight (g) | Thymus | Spleen | Kidney | Testes |
|---|---|---|---|---|---|---|
| Control | 10 | 25.9 ± 2.1[b] | 1.84 ± 0.30 | 3.12 ± 0.45 | 12.4 ± 0.7 | 8.15 ± 0.87 |
| Methyl-CCNU ($mg\,kg^{-1}$): | | | | | | |
| 55 | 4 | 16.8 ± 2.8[c] | 0.57 ± 0.11[c] | 1.68 ± 0.64[c] | 12.0 ± 1.9 | 3.66 ± 0.98[c] |
| 50 + 5; 1 day[d] | 5 | 16.7 ± 2.1[c] | 0.35 ± 0.16[c] | 2.72 ± 0.99 | 13.6 ± 1.4 | 3.72 ± 1.22[c] |
| 5 + 50; 1 day | 10 | 20.0 ± 1.1[ce] | 1.12 ± 0.78[ce] | 2.67 ± 0.52 | 11.7 ± 1.2 | 4.00 ± 1.23[c] |
| 50 + 5; 2 days | 4 | 16.8 ± 2.1[c] | 0.52 ± 0.27[c] | 1.98 ± 0.33[c] | 12.6 ± 1.4 | 4.40 ± 0.67[c] |
| 5 + 50; 2 days | 10 | 20.3 ± 1.8[ce] | 1.02 ± 0.21[ce] | 3.82 ± 1.08[e] | 11.7 ± 3.6 | 4.05 ± 0.79[c] |

[a]$n$ = number of mice examined; [b]Values are mean ± s.d.; [c]Significantly different from untreated controls ($P \leq 0.05$); [d]Time interval between subsequent treatments; [e]Significantly different from corresponding high-low dose combination ($P \leq 0.05$).

*The effect of low dose of methyl-CCNU administered prior to a lethal dose of melphalan in normal mice* In this experiment low doses (5 and $10\,mg\,kg^{-1}$) of methyl-CCNU were given 1, 2 or 3 days prior to a lethal dose ($20\,mg\,mg^{-1}$) of melphalan, which when given alone caused 100% mortality within 7 days (Figure 2). Mice pretreated with low doses of methyl-CCNU showed reduced mortality rate. When given one day apart, a 42% survival was evident in the mice primed with $5\,mg\,kg^{-1}$ methyl-CCNU. When the time interval was extended to 2 days the best effect (50% survival) was obtained when using $10\,mg\,kg^{-1}$ as a priming dose. Priming with 5 or $10\,mg\,kg^{-1}$ 3 days before melphalan had only a slight effect on survival. Based on these results, the effect of priming of mice with $10\,mg\,kg^{-1}$ methyl-CCNU 2 days before treatment with $15\,mg\,kg^{-1}$ melphalan was studied. Results of this experiment (data not shown) showed that this treatment increased the survival (13/20 in melphalan alone group *vs.* 20/22 in methyl-CCNU primed mice) and enhanced the recovery of thymus weight. Corrected thymus wet weights were $1.95 \pm 0.43$, $0.73 \pm 0.46$ and $1.13 \pm 0.33$ in control, melphalan treated and methyl-CCNU primed and melphalan treated mice, respectively.

*The effect of low-high dose combinations of methyl-CCNU in the treatment of Lewis lung carcinoma*

Since priming of mice with low doses of methyl-CCNU showed a protective effect against lethal doses in healthy mice, it was of interest to test whether high doses of methyl-CCNU would be also well tolerated in mice previously implanted with a tumour, and whether a therapeutic gain might be achieved with low-high dose regimens. This could happen if normal tissues would be protected by the priming low dose while the tumour would not (Millar *et al.*, 1982). We first used doses of methyl-CCNU which were higher than those previously used by us in experimental tumour systems (Peled *et al.*, 1982; Perk *et al.*, 1974; 1977). Mice were implanted s.c. with $1 \times 10^6$ 3LL tumour cells and treated 10 days later (when tumour size was $\sim 0.5$–$1.0\,cm^3$) with 45 or $40\,mg\,kg^{-1}$ methyl-CCNU, and combinations of 40 and 5 (high-low) and 5 and $40\,mg\,kg^{-1}$ (low-high). It appeared, however, that these doses were toxic: 30–60% of the treated mice died without tumours 6–25 days following treatment with methyl-CCNU. Mortality rate was significantly decreased with the low-high combination as compared to the high-low dose combination and to treatment with $45\,mg\,kg^{-1}$ (which caused the highest mortality), but was not different from that observed in mice with $40\,mg\,kg^{-1}$ methyl-CCNU. Overall, there was no beneficial effect of priming with the low dose on tumour growth. We then tested the effect of $35\,mg\,kg^{-1}$ methyl-CCNU as the high, tumour killing dose, and the low-high dose combinations of 5 and $35\,mg\,kg^{-1}$ administered 2 days apart. Results of this experiment are illustrated in Figure 3. All the untreated tumour inoculated mice died within 30 days (MST was 18 days). In contrast, mice treated with $35\,mg\,kg^{-1}$ or with $35\,mg\,kg^{-1}$ followed 2 days later by $5\,mg\,kg^{-1}$ showed longer survival (MST was 35 and 37 days, respectively). Following a temporary shrinking of the tumours for $\sim$11–18 days a new burst of tumour growth took place, which caused death within 34–58 days following tumour inoculation. The most pronounced effect was observed in the low-high dose group with MST of 44 days. However, even in this group 10/12 mice died within 60 days after tumour inoculation, with lung metastases in most animals and, occasionally, metastases in other organs (i.e. kidney, liver).

*Double treatment with low and high dose combinations of methyl-CCNU*

In two experiments mice were treated twice with high dose of methyl-CCNU and with low-high and high-low dose combinations. The time interval between the high and low doses was two days. In the first experiment an initial high dose of $30\,mg\,kg^{-1}$ methyl-CCNU was administered on day 9 and a second high dose on day 20 following s.c. inoculation of $2 \times 10^5$ 3LL tumour cells. A low dose ($5\,mg\,kg^{-1}$) was administered 2 days before or after the high dose. In this experiment there was no difference between the low-high *vs.* the high-low combinations until day 40, but survival at 50 days was 2/10 in the low-high combination as compared to 6/10 in the high-low dose combination. At 60 days there were 2/10 survivors in both groups. Rate of death was much faster in the group treated twice with the high dose alone; MST was 30 days as compared to 41 and 44 days in the low-high and high-low dose combinations, respectively. All the untreated tumour-inoculated control mice died within 20 days following inoculation. This experiment was then repeated using $30\,mg\,kg^{-1}$ as the high dose, given 7 and 18 days after tumour inoculation, and either 5 or $10\,mg\,kg^{-1}$ methyl-CCNU as the low dose. Results of this experiment (Table III) showed decreased toxicity, decreased rate of tumour development and increased lifespan in the low-high dose treated mice receiving $5\,mg\,kg^{-1}$, as compared to all other drug treatments which showed a similar increase in lifespan. Mortality in this experiment was mostly due to toxicity until day 46. Non-treated tumour-inoculated mice died $18 \pm 2$ days after tumour inoculation. Treatment with high dose of methyl-CCNU plus vehicle administration 2 days afterwards served as control. At 90 days there were three survivors in the group primed with $5\,mg\,kg^{-1}$ and one survivor in the group primed with $10\,mg\,kg^{-1}$ methyl-CCNU, and all these mice were without tumours on macroscopic and microscopic examination.

**Figure 2** Percent survival of mice pretreated with low dose ($5\,mg\,kg^{-1}$ ●——● and $10\,mg\,kg^{-1}$ ○——○) of methyl-CCNU at 1, 2 or 3 days prior to the administration of a lethal dose ($20\,mg\,kg^{-1}$) of melphalan. There were 10 to 12 mice/group. None survived the treatment with melphalan alone.

**Figure 3** Mortality rate of mice implanted s.c. with Lewis lung carcinoma cells and treated with low and high dose combinations of 5 and $35\,mg\,kg^{-1}$ of methyl-CCNU. There were 12 mice/group. The time interval between the low and high dose administration was 2 days.

**Table III** Summary of results on toxic deaths and tumour development in mice treated twice with high dose of methyl-CCNU and with low-high and high-low dose combinations

| | *Days after tumour inoculation* | | | |
|---|---|---|---|---|
| *Treatment* | *18* | *32* | *46* | *90* |
| None | 3/10 (3)[a] | 0 | 0 | 0 |
| | 2.0–3.0[b] | | | |
| Methyl-CCNU ($mg\,kg^{-1}$): | | | | |
| 30 | 10/10 (4) | 4/10 (3) | 3/10 (3) | 0 |
| | 0.1–0.4 | 0.2–1.5 | 0.2–2.2 | |
| 30 + vehicle | 10/10 (6) | 5/10 (2) | 2/10 (2) | 0 |
| | 0.1–0.4 | 1.0–2.0 | 0.3–0.5 | |
| 5 + 30 | 10/10 (1) | 7/10 (0) | 3/10 (0) | 3/10 (0) |
| | 0.1 | — | — | — |
| 10 + 30 | 10/10 (0) | 2/10 (0) | 1/10 (0) | 1/10 (0) |
| | — | — | — | — |
| 30 + 5 | 10/10 (2) | 1/10 (0) | 0 | 0 |
| | 0.1 | — | | |
| 30 + 10 | 9/10 (4) | 0/10 — | 0 | 0 |
| | 0.1–0.2 | — | | |

[a]Number of survivors/total number of mice examined (number of mice with palpable tumours is given in parentheses); [b]Range of tumour wet weight (g).

### Discussion

The results herein show that priming with low doses of methyl-CCNU at appropriate times before treatment with a high dose can reduce its toxicity in normal and in 3LL tumour bearing mice, and that this protocol does not protect the tumour. Thus, 5 or 10 $mg\,kg^{-1}$ methyl-CCNU administered 1 or 2 days prior to lethal doses, markedly decreased mortality, enhanced body weight gain, thymus and kidney weight and the normal morphology of these organs. Moreover, this treatment was beneficial when employed with lethal doses of melphalan, another alkylating agent and widely used anti-cancer drug. Also, and most important, the combination of low and high doses of methyl-CCNU appeared to have a therapeutic gain in mice bearing the rapidly proliferating and highly metastatic 3LL tumour. Even in experiments with Lewis lung carcinoma in which lifespan was not significantly increased by priming with a low dose, this treatment was not worse than the administration of high dose alone. There was evidence for a decrease in toxic deaths in tumour-bearing mice treated with the low-high dose combinations whenever the high doses administered alone were lethal, this in either single or double treatment modalities. There was no protection of the tumour by priming with low doses of methyl-CCNU. This phenomenon of a beneficial effect of low doses of cytotoxic anti-cancer drugs administered prior to high doses or prior to therapeutic or lethal doses of gamma irradiation, was previously reported by Rose *et al.* (1975) and by others (Gregory *et al.*, 1971; Blackett & Aguado, 1979; Millar & Hudspith, 1976; Millar *et al.*, 1975; 1978*b*, *c*, *d*; 1980). No mechanistic explanation for this effect has been elucidated yet. It was suggested that DNA precursors which are released from dead cells are involved (Millar *et al.*, 1978*a*). Since in sheep and man (Millar *et al.*, 1978*d*; Hedley *et al.*, 1978) the beneficial effect persists and is actually optimal 7 days after such treatment, when cellular breakdown products are probably not present in large quantities, one should postulate another mechanism. Since the protective effect of low doses of cytotoxic drugs against radiation damage and death was demonstrated for a variety of drugs, with different pharmacokinetic behaviour, one may consider the role of changes in drug metabolism by microsomal enzymes and thus the availability of drugs or their active metabolites to target tissues (i.e. bone marrow, intestinal epithelium) (Conney, 1965; Orrenius *et al.*, 1969; Hill *et al.*, 1975; Oliverio, 1976). BCNU, a nitrosourea compound and anticancer drug was shown to alter the activity of various microsomal enzyme systems (Wilson & Larson, 1981). This occurred, however, 20 days after treatment.

Although the nitrosoureas and other alkylating agents are considered to be cell non-specific, the DNA synthetic phase is the most sensitive to their action (Tannock, 1978). What effect do low doses of such drugs have on the progression of stem cells and proliferating cells through phases of the cell cycle is not known. Such treatment may cause the clustering of some cells in a phase of the cell cycle which is more resistant to high doses of the drug administered a few days afterwards (Kobayashi *et al.*, 1981). Millar *et al.* (1978*b*, *c*) have shown that the protective effect of priming with low doses of cyclophosphamide (CY) prior to high doses of CY or radiation is due to faster recovery of bone marrow stem cells and not to a reduction in the fraction of stem cells killed. However, it is difficult to distinguish between true stem cells and cells capable of replication and tissue 'regeneration' following an acute damage (Potten *et al.*, 1979). Improvement in stem cell survival is more difficult to demonstrate, as compared to the recovery of more mature cell populations. Priming with low doses of alkylating agents such as CY and nitrosoureas, may kill maturing cells (i.e. in the bone marrow) thus releasing stem cells from their inhibitory control (Fried *et al.*, 1973). One should note that priming with low doses of CY was also shown to protect slowly proliferating cells of the urothelium and the lung (Millar *et al.*, 1978*a*; Collis *et al.*, 1980; Evans *et al.*, 1983*b*). If cell recovery in both rapidly and slowly dividing cells is indeed the main event underlying the effect of low doses of CY, methyl-CCNU and other drugs, one should analyse repair enzymes, processes, following such treatment. Priming with low doses of CY was shown to be beneficial in the treatment of patients with melanoma (Hedley *et al.*, 1978) and in the treatment of human neoplasms transplanted into immunodeprived mice (Evans *et al.*, 1983*b*; 1984). The low-high dose combinations of nitrosoureas may be especially suitable in cancer patients. These compounds are very efficient tumour cell killing drugs (Valeriote *et al.*, 1968) which are, however, very toxic and are therefore used with long time intervals – 6 to 8 weeks – between subsequent treatments (Carter & Livingston, 1982). Administration of low dose of nitrosoureas prior to the high doses should be feasible timewise.

A recent report on the inconsistency of the effect of priming with low doses of CY prior to high doses of melphalan in mice (Kulkarni *et al.*, 1985) seems to point to potential difficulties in adopting this approach to man. However, the multitude of data pointing to a beneficial effect of such treatment in tumour bearing mice, including those transplanted with human tumours, calls for further studies on the effect of low doses of cytotoxic drugs on different cell populations, according to their stage of differentiation and maturation, and on recovery processes and drug metabolism.

### References

BLACKETT, N.M. & AGUADO, M. (1979). The enhancement of haemopoietic stem cell recovery in irradiated mice by prior treatment with cyclophosphamide. *Cell Tissue Kinet.*, **12**, 291.

CARTER, S.K. & LIVINGSTON, R.B. (1982). Drugs available to treat cancer. In *Principles of Cancer Treatment*, Carter, S.K. *et al.* (eds) p. 111. McGraw-Hill Book Company: New York.

COLLIS, C.H., WILSON, C.M. & JONES, J.M. (1980). Cyclophosphamide induced lung damage in mice: Protection by a small preliminary dose. *Br. J. Cancer*, **41**, 901.

CONNEY, A.H. (1965). Enzyme induction and drug toxicity. In *Drugs and Enzymes*, Brodie, B.B. & Gilette, F.R. (eds) p. 277. The Macmillan Company: New York.

EVANS, B.D., SMITH, I.E. & MILLAR, J.L. (1983a). High dose cyclophosphamide treatment of human oat cell xenografts in immune deprived mice. *Br. J. Cancer,* **47,** 215.

EVANS, B.D., SMITH, I.E., CLUTTERBUCK, R.D. & MILLAR, J.L. (1983b). Normal tissue toxicity and antitumour experiments carried out in mice using high-dose cyclophosphamide. *Cancer Treat. Rev.,* **10,** Suppl. A, 25.

EVANS, B.D., SMITH, I.E., CLUTTERBUCK, R.D. & MILLAR, J.L. (1984). Prevention of acute deaths in mice after very high dose cyclophosphamide by divided dose schedule. *Br. J. Cancer,* **49,** 43.

FRIED, W., HUSSEINI, S., GREGORY, S., KNOSPE, W.H. & TROBAUGH, F.E., JR. (1973). Effect of cyclophosphamide on the hematopoietic microenvironmental factors which influence hematopoietic stem cell proliferation. *Cell Tissue Kinet.,* **6,** 155.

GREGORY, S.A., FRIED, W., KNOSPE, W.H. & TROBAUGH, F.E. JR. (1971). Accelerated regeneration of transplanted hematopoietic stem cells in irradiated mice pretreated with cyclophosphamide. *Blood,* **37,** 196.

HEDLEY, D.W., MILLAR, J.L., MCELWAIN, T.J. & GORDON, M.Y. (1978). Acceleration of bone marrow recovery by pretreatment with cyclophosphamide patients receiving high-dose melphalan. *Lancet,* **ii,** 966.

HILL, D.L., KIRK, M.C. & STRUCK, R.F. (1975). Microsomal metabolism of nitrosoureas. *Cancer Res.,* **35,** 296.

KOBAYASHI, S., HOSHINO, T. & WILSON, C.B. (1981). Time-dependent synergism of low-dose 5-fluorouracil (5-FU) with hydroxyurea (HU) in the treatment of 9L cells *in vitro. Proc. Am. Cancer Assoc.,* **22,** 208.

KULKARNI, S.S., LEVENTON, G.S., HUYNH, L., CHOW, H., DICKE, K.A. & ZANDER, A.R. (1985). Effect of pretreatment with cyclophosphamide on high-dose toxicity of melphalan in mice. *Cancer Res.,* **45,** 5431.

MILLAR, J.L., HUDSPITH, B.N. & BLACKETT, N.M. (1975). Reduced lethality in mice receiving a combined dose of cyclophosphamide and busulphan. *Br. J. Cancer,* **32,** 193.

MILLAR, J.L. & HUDSPITH, B.N. (1976). Sparing effect of cyclophosphamide (NSC-76271) pretreatment on animals lethally treated with γ-irradiation. *Cancer Treat. Rep.,* **60,** 409.

MILLAR, J.L., BLACKETT, N.M. & HUDSPITH, B.N. (1978a). Enhanced post-irradiation recovery of the haemopoietic system in animals pretreated with a variety of cytotoxic drugs. *Cell Tissue Kinet.,* **11,** 543.

MILLAR, J.L., HUDSPITH, B.N., MCELWAIN, T.J. & PHELPS, T.A. (1978b). Effect of high-dose melphalan on marrow and intestinal epithelium in mice pretreated with cyclophosphamide. *Br. J. Cancer,* **38,** 137.

MILLAR, J.L. & MCELWAIN, T.J. (1978c). Combinations of cytotoxic agents that have less than expected toxicity on normal tissues in mice. *Antibiot. Chemother.,* **23,** 271.

MILLAR, J.L., PHELPS, T.A., CARTER, R.L. & MCELWAIN, T.J. (1978d). Cyclophosphamide pretreatment reduces the toxic effect of high-dose melphalan on intestinal epithelium in sheep. *Eur. J. Cancer,* **14,** 1283.

MILLAR, J.L., CLUTTERBUCK, R.D. & SMITH, I.E. (1980). Improving the therapeutic index of two alkylating agents. *Br. J. Cancer,* **42,** 485.

MILLAR, J.L., STEPHENS, T.C. & WIST, E.A. (1982). An explanation for the ability of cytotoxic drug pretreatment to reduce bone marrow related lethality of total body irradiation (TBI). *Int. J. Radiat. Oncol. Biol. Phys.,* **8,** 581.

OLIVERIO, V.T., (1976). Pharmacology of the nitrosoureas: An overview. *Cancer Treat. Rep.,* **60,** 703.

ORRENIUS, S., DAS, M. & GNOSPELLIUS, Y. (1969). Overall biochemical effects of drug induction on liver microsomes. In *Microsomes and Drug Oxidations,* Gillette, J.R. *et al.* (eds) p.251. Academic Press: New York.

PELED, A., PERK, K., HARAN-GERA, N. & CHIRIGOS, M.A. (1982). The oncostatic effect of methyl-CCNU on various experimental lymphoreticular neoplasms. *Leukemia Res.,* **6,** 89.

PERK, K., PEARSON, J.W., TORGERSEN, J.A. & CHIRIGOS, M.A. (1974). An animal model for meningeal leukemia. *Int. J. Cancer,* **13,** 863.

PERK, K. & PEARSON, J.W. (1977). MeCCNU in the treatment of an animal analog of meningeal leukemia. *Israel J. Med. Sci.* **13,** 460.

POTTEN, C.S., SCHOFIELD, R. & LAJTHA, L.G. (1979). A comparison of cell replacement in bone-marrow, testis and three regions of surface epithelium. *Biochim. Biophys. Acta,* **560,** 281.

ROSE, W.C., RIMM, A.A., SALZSTEIN, E.C., TRUITT, R.L. & BORTIN, M.M. (1975). Low-dose chemotherapy as a prelude to intensive treatment of spontaneous leukemia-lymphoma in AKR mice. *J. Natl Cancer Inst.,* **55,** 219.

SNEDECOR, G.W. & COCHRAN, W.G. (1967). *Statistical Methods,* 6th ed. Iowa State University Press: Ames.

TANNOCK, I. (1978). Cell kinetics and chemotherapy: A critical review. *Cancer Treat. Rep.,* **62,** 1117.

VALERIOTE, F.A., BRUCE, W.R. & MEEKER, B.E. (1968). Synergistic action of cyclophosphamide and 1,3-bis(z-chlorethyl)-1-nitrosourea on a transplanted murine lymphoma. *J. Natl Cancer Inst.,* **40,** 935.

WILSON, V.L. & LARSON, R.E. (1981). Delayed alterations in hepatic mixed function oxygenase enzymes in Carmustine treated mice. *Proc. Am. Assoc. Cancer Res.,* **22,** 38 (*Abstract*).

ZIMBER, A. & PERK, K. (1978). Male sterility in the domestic fowl (*Gallus domesticus*) after 1-(2-chloroethyl)-3-(4-methylcyclohexyl)-1-nitroso-trans-urea (NSC-95441) treatment of chicks. *J. Natl Cancer Inst.,* **61,** 793.

ZIMBER, A. & PERK, K. (1979). The effect of early treatment with methyl-CCNU on the lymphoid organs and the humoral immune response in chickens. *Poultry Sci.,* **58,** 162.

ZIMBER, A., YEGANA, Y. & PERK, K. (1980). Some pathophysiological aspects of methyl-CCNU treatment in mice and rats. *Refuah Vet.,* **37,** 60 (*Abstract*).